# Short-Term Impact of Newly Imposed Legal Restriction on DSD Surgery in Children in Germany

**DOI:** 10.3390/children11091104

**Published:** 2024-09-09

**Authors:** Frank-Mattias Schäfer, Benjamin Schwab-Eckhardt, Egbert Voß, Michael Schroth, Franz Staudt, Maximilian Stehr

**Affiliations:** 1 Department of Pediatric Surgery and Pediatric Urology, Cnopfsche Kinderklinik, 90419 Nürnberg, Germany; benjamin.schwab-eckhardt@diakoneo.de (B.S.-E.); maximilian.stehr@diakoneo.de (M.S.); 2 Clinic of Urology and Pediatric Urology, University Hospital Erlangen, Friedrich-Alexander-Universität (FAU) Erlangen-Nürnberg, 91054 Erlangen, Germany; 3 Department of Pediatric Endocrinology, Cnopfsche Kinderklinik, 90419 Nürnberg, Germany; egbert.voss@diakoneo.de; 4 Department of Pediatrics and Neonatology, Cnopfsche Kinderklinik, 90419 Nürnberg, Germany; michael.schroth@diakoneo.de; 5 Master in Ethics, 94036 Passau, Germany; franz.staudt@t-online.de

**Keywords:** DSD, disorders of sexual development, congenital adrenal hyperplasia, hypospadias

## Abstract

Background/Objectives: In recent years, changing paradigms, both culturally and scientifically, have fundamentally altered the approach to the treatment of children with Disorders of Sexual Development (DSD) prior to reaching the age of legal consent. In Germany, the situation changed with the introduction of legislation that includes a partial ban on DSD surgery in children in 2021. This study aims to analyze the impact of this legislation on clinical practice. Methods: From 2014 to 2024, all patients with DSD in our institution were included. The study group comprised all patients operated on after the legislation. All patients operated on before the legislation served as the control group. Karyotype, phenotype, resulting type of DSD, age at presentation and age at operation were recorded. Results: A total of 35 patients were included in this study, with 15 in the study group and 20 in the control group. The operation was authorized by the family court for all patients in the study group. A total of 46,XY patients with severe hypospadias and clinical aspect of intersexual outer genitalia were the largest proportion (25 patients, 71.4%). Nine patients (25.7%) were 46,XX girls with classical congenital adrenal hyperplasia (CAH) type. One patient (2.9%) showed a mixed gonadal dysgenesis. The mean age of the patients at first presentation in our institution was 10.7 months in the control group and 11.0 months in the study group. The mean age at operation was significantly higher in the study group (20.1 months) compared to the control group (15.1 months; *p* = 0.032, unpaired *t*-test). Conclusions: The introduction of the legislation with a partial ban of genital surgery in DSD children in Germany has led to a significant delay in surgery. Since the majority of the patients comprise severe hypospadias and 46,XX CAH patients, further amendments of the law are proposed to minimize potential harm.

## 1. Introduction

In recent years changing paradigms, both culturally and scientifically, have fundamentally altered the approach to the treatment of children with Disorders of Sexual Development (DSD) prior to the age of ability to legal consent, especially in western countries. The question “if (why), when, and how?” has sparked a marked discussion both in the scientific community and in the broader society [1]. It is remarkable that there is no fundamental agreement even on the question of which diagnoses fall under the acronym DSD. From a strict scientific standpoint, the most widely accepted classification is the Chicago Consensus classification from 2006 [2].

While not all patients with DSD according to this classification potentially require gender reassignment surgery due to the appearance of their outer genitalia, there are several groups in which the importance of this issue is more pronounced, and parents usually seek advice on how to harmonize outer genitalia with the child’s karyotype. This is usually the case for patients in the 46,XX-DSD group with classical congenital adrenal hyperplasia (CAH), which feature a varying degree of virilized outer genitalia up to a normal-looking phallus with female inner genitalia and no testes. This appearance resembles the outer genitalia features of a 46,XY patient with severe hypospadias with non-palpable testes [3]. For this group of patients, there is increasing evidence that 46,XX classical CAH patients do not want to be considered DSD patients [4]. The group of 46,XY-DSD patients consists of a very heterogeneous set of disorders or situations, in most of which surgery is not a central issue [1]. However, testicular retention can be a situation which may warrant surgery in some conditions and in this group, severe hypospadias (without further differentiation) are also included, which is usually a condition where surgical correction is warranted early in life.

The groups with the greatest variability are patients with sex-chromosome mosaics and ovo-testicular DSD, as they can have a broad variability in appearance of the outer genitalia ranging from a normal male phenotype to a mixture of testicular and ovarian tissue e.g., with a severe hypospadic appearance. This variability warrants a highly individualized approach and raises difficult questions regarding both gender assignment and genital reconstruction, as well as their timing [1].

In anticipation of the legislative process, it has been stated in the first interdisciplinary German consensus guideline that specific surgical procedures should be postponed in DSD patients until the individuals can make their own decision as to what kind of surgery the patient prefers. Only in cases of vital harm and a strictly somatic functional or vital medical indication may surgical procedures be conducted on minors [5].

Interestingly, the patient representative group for CAH patients gave their dissent to this consensus guideline, insofar as they rejected a restriction of gender assignment based on a phenotypical variation of the outer genitalia due to a metabolic disorder during pregnancy and an unequivocal female inner genitalia and female genotype. They termed the planned legislation a “pretention of protection” [5].

Nevertheless, in Germany, the legislation process started with the coalition treaty after the federal election in 2017. This coalition agreement called for a legal prohibition of gender reassignment surgery on children unless it cannot be postponed and is necessary to avert danger to life. After a lengthy process involving several stakeholder groups and medical experts, the law came into force on 25 May 2021: The newly introduced paragraph 1631e of the German Civil Code (Bürgerliches Gesetzbuch, BGB) states that parental personal care and thus the right of consent of the parents of a child with DSD does not include the right to treatment if this “is carried out solely with the intention of harmonizing the physical appearance of the child with that of the male or female sex”. In view of the discussion outlined above, however, consent is possible if the surgical interventions, which “could result in an alignment … with that of the male or female sex”, cannot “be postponed until the child has made a self-determined decision”.

The law stipulates that these interventions must always be authorized by a family court decision prior to surgery (except in emergency situations). While non-syndromic undescended testes and distal hypospadias are therefore not subject to authorization by the family court, as they are not classified as DSD by the Chicago Consensus classification, this is different for patients with “severe hypospadias”, as they are classified as a subtype of 46,XY-DSD in the said Consensus classification. Because the Chicago Consensus classification was never meant to be the basis for a legislative process but to completely describe the different forms of DSD patients from a purely scientific standpoint, this leaves room for interpretation and confusion.

To address this, the legislature has further stipulated that “authorization shall be granted at the request of the parents if the planned intervention is in the best interests of the child. If the parents submit to the family court an opinion in favor of the intervention from an interdisciplinary commission …, it shall be assumed that the planned intervention is in the best interests of the child”.

Since then, in our institution operations in children with diagnosis falling under the DSD classification according to the Chicago Consensus have been performed following the legal procedures mentioned above.

The aim of this study was to analyze the changes in the surgical approach in these patients and, in particular, to investigate whether the legislation has led to a significant delay in surgery.

## 2. Materials and Methods

Patient selection: After obtaining approval from the institutional ethics committee, all patients who sought advice in our institution with children with any form of DSD in accordance with Section 1631e of the German Civil Code were included in this study. This applies to all patients who underwent surgery after 25 May 2021 (when the law came into force) until 1 July 2024. All these patients had to undergo family court approval before surgery.

As a comparison group, all patients who underwent an operation between 1 January 2014 and 24 May 2021 (before the law came into force) were included. According to the wording of the law, a corresponding family court consent would have been necessary if the law had already been in force at that time.

For the analysis, each patient’s diagnosis, i.e., type of DSD according to the Chicago classification, was noted. Therefore, both karyotype and the results of additional molecular genetic tests, such as for steroid biosynthesis defects etc. (if performed), were recorded. For further analysis the age at first presentation at our institution (where they received multidisciplinary advice on how to proceed), the date of the interdisciplinary expert opinion, the date of the court decision and the patient’s age at the time of surgery were recorded. For the purpose of this study, in cases of staged surgery (such as in scrotal or perineal hypospadias), the time of the first stage of the correction was used.

As stated above, the use of the Chicago Consensus classification as a basis for legal consideration has its own limitations. From a practical approach, in 46,XY-DSD the term “severe hypospadias” was therefore defined as any hypospadias preoperatively described as penoscrotal, scrotal or perineal hypospadias with or without testicular retention and concomitant penoscrotal transposition and bifid scrotum, resulting in the clinical appearance of an ambiguous genitalia. Chromosomal mosaics and 46,XX-DSD with severely virilized outer genitalia (Prader IV–V) were also included in this study. However, for the purpose of this study, patients with severe “non-hormonal/non-chromosomal DSD”, such as cloacal exstrophy, were not included in this study, as the first surgery is not primarily intended to harmonize the outer genitalia of the child to a male or female appearance, and therefore family court approval is considered unnecessary in cases where the creation of hindgut stoma and bladder closure are imminent.

Genetic and endocrinological testing: All patients received karyotype testing before counselling. Endocrinological examinations for mineral and glucocorticoid precursors, cortisol, androstendione, testosterone and dehydroepiandrosterone (DHEAS) were performed routinely after birth. Patients with suspicion of CAH received adequate molecular genetic testing, in particular for 21-hydroxylase deficiency, 11ß-hydroxylase deficiency and 3ß-hydroxysteroid dehydrogenase deficiency. Additional genetic testing was performed as deemed necessary by the primary pediatric endocrinologist. All patients presented with a final diagnosis and a complete set of genetic and endocrinologic testing in our pediatric urology department.

Interdisciplinary commission’s opinion for family court approval: According to the law, for all patients treated after the law came into effect, an interdisciplinary commission is formed to prepare an expert opinion for the family court responsible for the individual patient. This interdisciplinary commission must include the following persons/specialties: (a) the person treating the child, (b) a person who has a professional qualification in psychology, child and adolescent psychotherapy or child and adolescent psychiatry, (c) a person who has earned a degree in ethics. Additionally, the medical committee members must have different pediatric specializations. They must include a specialist in pediatric and adolescent medicine with a focus on pediatric endocrinology. One member of the commission may not be employed in the medical care facility in which the surgical procedure is to be performed. All members of the commission must have experience in dealing with children with variations in gender development. At the request of the parents, the commission shall involve a counsellor with a gender developmental variant (i.e., an adult patient with an appropriate type of DSD).

The opinion of the interdisciplinary commission in favor of the surgical intervention must contain the following information in particular: (a) why the commission is in favor of the intervention, taking into account the best interests of the child, and whether it considers it to be in the best interest of the child, in particular which risks are associated with the proposed intervention, with other possible treatments or with refraining from an intervention until the child is able to make a self-determined decision, (b) whether and by which members of the commission a discussion was held with the parents and the child (if possible) and whether and by which members of the commission the parents and the child were informed and counselled on how to deal with this variant of gender development, (c) whether counselling of the parents and the child by a counsellor with a variant of gender development has taken place, (d) to what extent the child is able to form and express an opinion and whether the planned intervention is in accordance with the child’s wishes, as well as (e) whether the counsellor with a variant of gender development involved supports the favorable opinion.

After coming to a conclusion, the committee’s recommendation is then forwarded to the parents, who must then apply to the local family court for authorization of the surgical treatment. After approval, the operation can be performed.

In our institution, in cases of severe hypospadias, correction is usually performed in a two-stage approach [6], while feminizing genitoplasty of virilized outer genitalia in CAH patients is usually corrected in a one-stage approach, including urogenital mobilization, clitoral reduction with preservation of the neurovascular bundle, labioplasty by fashioning labia minora via creating Byars flaps by splitting the dorsal prepuce skin and labia majora by using the labioscrotal skin [7,8,9]. For the purpose of this study, in cases of two-stage operations, the first step of the correction was used as the endpoint.

Statistical Analysis: Data processing and calculation were performed using GraphPad Prism^®^ 10.2.2 (GraphPad Software Inc, Boston, MA, USA) and Microsoft Excel^®^ 365 (Microsoft Corporation, Redmond, WA, USA). An unpaired *t*-test, Fisher’s exact test and the chi-square test were used to analyze the data. An estimation plot was used to present the magnitude of the effect on the age at surgery, which allows a visual representation of its precision (confidence interval). *p* values < 0.05 were considered statistically significant.

## 3. Results

### 3.1. Patient Demographics

A total of 35 patients were included in this study: 15 of the patients after the legal restriction became effective and 20 in the period before the introduction of the law.

The characterization of patients according to a simplified 2005 Chicago Consensus classification scheme is given in Table 1. It can be seen that the patients treated were predominantly from very few groups of DSD. Interestingly, in the 46,XY group, no patients with androgen synthesis defect or androgen insensitivity syndrome were identified, which led to the inclusion of these patients into section C (“other”) of the 2005 Chicago Consensus classification.

**Figure 1:**
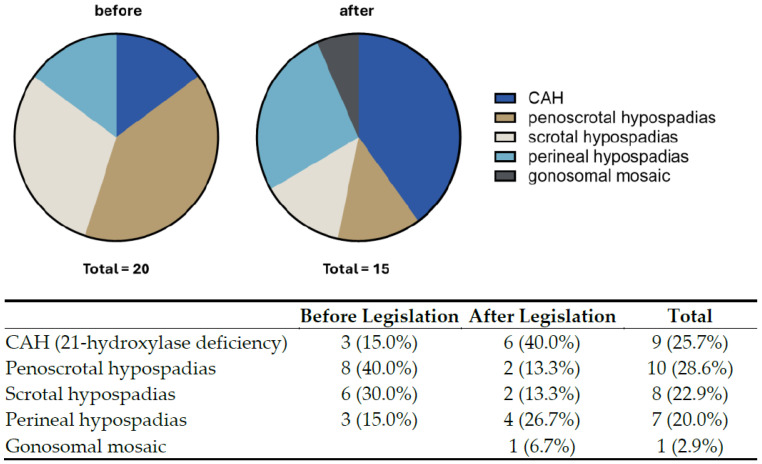
Graphic distribution of the patients’ DSD classification before and after the legislation. It is noteworthy that there was a tendency towards a higher proportion of CAH patients and increased severity of hypospadias after the legislation came into effect.

A detailed analysis of the patient characteristics before and after the legislation shows that there was a tendency towards a more severe phenotype in regards of hypospadias, as well as in increase in the rate of CAH patients after the legislation came into effect, although this was not statistically significant (Figure 1, *p* = 0.11, Fisher’s exact test). All patients showed either a 46,XY or and 46,XX karyogram except one, who showed a 45X/46X,idic(Y)(q11.2) karyotype: in addition to a cell line with a monosomy X, a cell line with an isodicentric Y chromosome was found. This was interpreted as a gonosomal mosaic DSD. This patient’s phenotype showed a scrotal hypospadias with one descended and one inguinal testis, with a bifid scrotum. A testicular biopsy had been taken in an outside hospital beforehand and showed normal pre-pubertal testicular tissue without any evidence of ovarian tissue.

All nine 46,XX-CAH patients had classical CAH with 21-hydroxylase deficiency and showed either Prader stage IV (six patients) or V (three patients).

Of note, the frequency of presentation changed markedly in the two periods: in the control group before the legislation came into force, the average rate was 2.7 patients per year, which increased to an average of 4.5 patients per year after the introduction of the law, almost doubling the number of patients per year.

### 3.2. Age at Presentation

The mean age of the patients at first presentation in our institution was 10.7 months in the control group (range 4.0 to 30.4 months) and 11.0 months (range 1.7 to 27.7 months). The difference was not statistically significant.

Of the 15 patients after the law was introduced, the parents of one patient with penoscrotal hypospadias and penoscrotal transposition chose not to have their children operated on and did not seek a family court decision. This patient was excluded from further analysis, as no further information is available (loss of follow-up). All other patients were operated on after family court approval was granted in all cases. In the control group, all caregivers chose to have their children operated on.

### 3.3. Age at Operation

**Figure 2:**
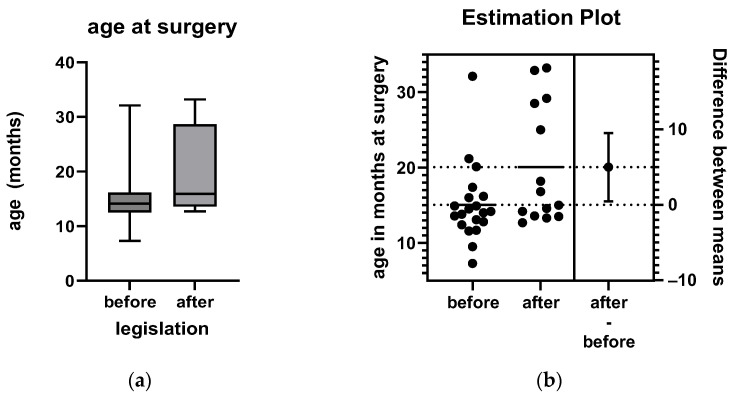
(**a**) Box and whiskers plot of the age at surgery before and after the legislation: the box represents the lower and upper quartiles, while the whiskers show the minimum and maximum age. (**b**) Estimation plot showing the increased age of surgery after imposing the restrictive law. The mean increase in age was 5.0 months (SEM ± 2.2 months: 95% CI interval: 0.47 to 9.51) compared to the control group.

In the control group, the mean age at the operation (for two-stage operations, the timing of the first stage) was 15.1 months (median 14.1 months; range: 7.3–32.1 months, SD 5.2 months). In the group after introduction of the legislation the mean age at operation was 20.1 months (median 15.9 months; range: 12.7–33.7 months, SD 7.9 months). The difference was statistically significant (*p* = 0.032; unpaired *t*-test). A detailed visualization of the differences is shown in Figure 2.

The proportion of patients operated on after the age of 18 months was 15.0% (3/20) in the control group and 42.9% (6/14) in the group after the introduction of the legislation. This tendency, however, was not significant (*p* = 0.11, Fisher’s exact test).

The mean duration of the legal process (the date of the interdisciplinary commission opinion to date of family court decision) was 2.6 months (range 0.5–5.8 months); however, this did not include the process of organizing and writing the commission’s opinion, which took place beforehand.

## 4. Discussion

To our knowledge, this study presents for the first time a series of patients treated in a legal context where operating on children with DSD before age of consent is heavily regulated by federal legislation. Germany is one of the first countries to introduce such a partial ban of gender reassignment surgery. Similar bans are already in place in other countries or regions of the world, including countries such as Malta and Portugal or states such as California [10,11]. Moreover, in February 2019, the Parliament of the European Union adopted a resolution condemning sex-normalizing surgery and encouraging the member states to adopt legislation addressing it [10]. Therefore, there is an increasing likelihood that more nations or regions will adopt similar legislation. There are also fears in other regions, such as India, that imposing laws or rules to ban all DSD surgeries may lead to an increased use of paramedical means, charlatanism and quackery, because the ban will not be accepted by large parts of the population due to different socio-cultural backgrounds [12].

This highlights the necessity of scientific data on the impact of such legislation. While long-term data are naturally not yet available, we are able to present some data on the short-term consequences after the introduction of the German regulative law for surgery in DSD children. Our data show a significant increase in the age at surgery, due to the necessary regulatory process until authorization by the family court is granted. This leads to an increase in the proportion of patients being older than 18 months at the operation from 15% to 43%.

While this in itself is not yet significant due to the still small patient numbers, the tendency shows that this delay may potentially be detrimental to patients. The current concept in severe hypospadias surgery (whether deemed isolated or as part of DSD) advocates early surgery, allowing for a staged repair and the reconstruction of potential complications, such as fistulas, prior to 18 months of age [13,14,15,16]. The situation is quite similar in classical 46,XX CAH patients, a condition that, as is universally accepted, is best repaired in infancy, especially in cases of severely virilization, as was the case for all patients in our study [7,17]. Therefore, a cutoff of 18 months as an appropriate time limit for surgery was chosen in our analysis for all patient groups.

Interestingly, the increase in the frequency of presentation of DSD patients in our institution after the legislation may point to a resulting centralization, as some hospitals or departments may have difficulties in creating the required interdisciplinary commission, managing the effort involved or dealing with the legal complexities. While centralization itself is a desirable goal in the treatment of these patients [18], there is also the possibility that some patients may be deprived of adequate treatment because they do not have access to a centralized treatment center and adequate legal advice. Naturally, no data exist on this question so far.

It is noteworthy that the two main groups in this study (46,XX-DSD patients with CAH and 46,XY-DSD patients with severe hypospadias) represent over 90% of the patient collective. The relatively small proportion of CAH patients may be explained by the fact that only patients with severe virilization (Prader IV-V) were included in this study, while the families of CAH patients with less severe virilization did not seek surgical correction.

In consequence, these two main groups of our study pose special problems in regard to the DSD legislation and, therefore, surgery under these conditions. As our data show, in all patients, surgery was granted because the multidisciplinary board recommendation argued that the operation would be in the patient’s best interest. Additionally, in 46,XX CAH patients, there is increasing scientific evidence that these patients do not want to be considered as intersex patients [19]. Therefore, both the German patient representative group and the US-American CARES (Congenital Adrenal Hyperplasia Research Education and Support) Foundation reject the characterization of their members as DSD patients [5,11]. On the contrary, CARES views CAH not as a sexual disorder at all, but as a life-threatening adrenal disorder [11]. It is therefore increasingly difficult to justify why these patients should be covered by surgery-restricting DSD legislation.

Furthermore, there is also another medical ethics issue to consider: from a medical-ethical point of view, it is not consistent to regard CAH patients as DSD patients and therefore to place the surgical *treatment* of the virilized outer genitalia under legal reservation, but not the *prevention* of virilization of the external genitalia in pregnancies of affected mothers by prenatal dexamethasone therapy [20]. Although early implementation of dexamethasone in the prenatal treatment of CAH has been controversial, there is recent evidence that this treatment can reduce long-term complications [21]. Therefore, from a medical–ethical point of view, leaving this matter to a legal decision seems questionable.

Several problems arise from the necessity of creating an interdisciplinary expert commission for children with DSD. Firstly, there is an administrative burden that is not reflected in the remuneration provided by health insurance funds. Secondly, the need to consult a qualified person with a corresponding variant of gender development cannot always be met. This is particularly the case for patients with severe hypospadias, as there is no recognized patient representative group for these patients in Germany (as there is for CAH patients, for example).

In our opinion, an appropriate solution for this complex situation would be to exempt both classical 46,XX CAH patients and severe 46,XY hypospadias patients from the legislation because, on the one hand, as far as we know, their operations are all approved by the family court anyway and, on the other hand, in the case of CAH patients, the affected families and patient/parent representative organizations are opposed to this legislation (and the underlying classification). In addition, most 46,XX CAH patients raised as girls have no gender dysphoria [1]. In these patients with classic form of CAH, a correct female gender assignment, a prompt surgical treatment with external genital ambiguity correction and an adequate disease treatment, with the control of androgen excess, permit an increased probability of maintaining general wellbeing, perceiving a female gender identity and better managing sexual dysfunctions, especially related to dyspareunia [22].

In our study, exempting patients with these two diagnoses would have reduced the workload for family court approval both for the courts and the other stakeholders by 93% (13 of 14 patients operated on after the legislation), while still allowing for an individual case-by-case decision in the much rarer and more controversial situations, such as gonosomal mosaic patients.

Strengths and limitations of this study: Part of the study period fell during the coronavirus pandemic, which led to delays in both outpatient visits and the planning of elective operations [23]. This may result in effects that cannot be quantified more precisely in the setting of this study. However, since the strictest lockdown policies occurred during the control group before the legislation, it is unlikely that the post-legislation group was more affected by these policies. In addition, our study comprised only a small series, with the majority of patients having CAH or severe hypospadias. The conclusion drawn may not be suitable for other complex situations in DSD patients with different diagnosis. The aim of this study was to assess the short-term consequences of the German legal restrictions on gender assignment surgery in children. It is therefore not yet possible to assess the long-term consequences. For this aim, a life-long follow-up in multi-center longitudinal studies, which should also include patients who are not operated on during childhood, is required to create this much-needed data [18]. However, this will be difficult to achieve. Possibly, national adaptations of international registers such as the I-DSD and I-CAH registers, reflecting the current legal situation, may be beneficial [18,24].

## 5. Conclusions

After the introduction of a partial ban of genital DSD surgery in children in Germany in 2021, the timing of the surgery increased significantly, leading to a mean delay of 5 months and therefore to an exceeding of the optimal time window for surgery in a considerable proportion of patients. This affected mostly 46,XX CAH patients and 46,XY hypospadias patients. Since family courts ruled in favor of surgery in all cases, consideration should be given to excluding these diagnoses in future amendments to the legislation.

## Figures and Tables

**Table 1:** Characteristics of DSD patients according to the 2005 Chicago Consensus classification.

Sex Chromosome DSD	46,XY-DSD	46,XX-DSD
A: 45, X (Turner syndrome and variants) *(none)*	A: disorders of gonadal (testicular) development *(none)*	A: Disorders of gonadal (ovarian) development, e.g., ovotesticular DSD *(none)*
B: 47, XXY (Klinefelter syndrome and variants) *(none)*	B: Disorders in androgen synthesis or action *(none)*	B: Androgen excess, e.g., 21-hydroxylase deficiency (classical CAH) ***(9 patients—25.7%)***
C: 45, X/46,XY (mixed gonadal dysgenesis, ovotesticular DSD) ***(1 patient—2.9%)***	C: Other (e.g., severe hypospadias) ***(25 patients—71.4%)***	C: Other (e.g., cloacal exstrophy) *(none/excluded)*
D: 46,XX/46,XY DSD (chimeric, ovotesticular DSD) *(none)*		

## Data Availability

The underlaying data presented in this study are available on request from the corresponding author due to restrictions in the Institutional Ethics Committee approval.
